# 4-[3-(Pyridin-4-yl)prop­yl]pyridinium 2-carb­oxy­benzoate

**DOI:** 10.1107/S1600536812029339

**Published:** 2012-07-04

**Authors:** Wei Xu, Jin-Li Qi

**Affiliations:** a Center of Applied Solid State Chemistry Research, Ningbo University, Ningbo 315211, People’s Republic of China

## Abstract

In the title molecular salt, C_13_H_15_N_2_^+^·C_8_H_5_O_4_^−^, the 2-carb­oxy­benzoate anions are joined into a chain along [010] by strong O—H⋯O hydrogen bonds, with the H atoms disordered about the inter­vening centres of inversion. The presence of N—H⋯O hydrogen bonds between cations generates an additional chain along [010] and parallel to that of the anions. The chains are assembled into a three-dimensional framework *via* weak C—H⋯O inter­chain inter­actions. In the cation, thee dihedral angle between the pyridine rings is 48.91 (4)°.

## Related literature

For the applications of co-crystals, see: Schultheiss & Newman (2009 ▶); Sarma *et al.* (2011 ▶). For the design of co-crystals, see: Callear *et al.* (2010 ▶); Braga *et al.* (2011 ▶). 
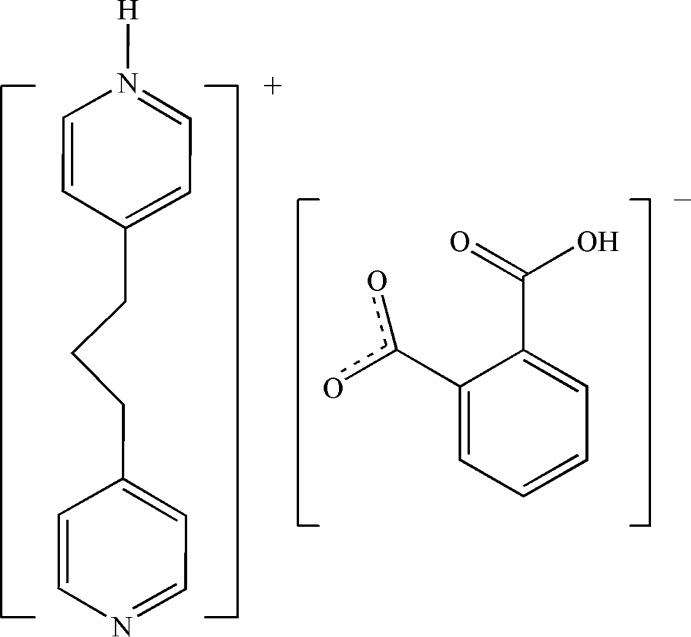


## Experimental

### 

#### Crystal data

C_13_H_15_N_2_^+^·C_8_H_5_O_4_^−^
*M* *_r_* = 364.39
Orthorhombic, 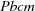

*a* = 7.5950 (15) Å
*b* = 12.822 (3) Å
*c* = 17.340 (4) Å
*V* = 1688.6 (6) Å^3^
*Z* = 4
Mo *K*α radiation
μ = 0.10 mm^−1^
*T* = 295 K
0.35 × 0.24 × 0.21 mm

#### Data collection

Rigaku R-AXIS RAPID CCD diffractometer
Absorption correction: multi-scan (*ABSCOR*; Higashi, 1995 ▶) *T*_min_ = 0.761, *T*_max_ = 0.865
16479 measured reflections
2097 independent reflections
1468 reflections with *I* > 2σ(*I*)
*R*_int_ = 0.039

#### Refinement

*R*[*F* ^2^ > 2σ(*F* ^2^)] = 0.042
*wR*(*F* ^2^) = 0.118
*S* = 1.14
2097 reflections
134 parameters
H-atom parameters constrained
Δρ_max_ = 0.25 e Å^−3^
Δρ_min_ = −0.24 e Å^−3^

### 

Data collection: *RAPID-AUTO* (Rigaku, 1998 ▶); cell refinement: *RAPID-AUTO*; data reduction: *CrystalStructure* (Rigaku/MSC, 2004 ▶); program(s) used to solve structure: *SHELXS97* (Sheldrick, 2008 ▶); program(s) used to refine structure: *SHELXL97* (Sheldrick, 2008 ▶); molecular graphics: *SHELXTL* (Sheldrick, 2008 ▶); software used to prepare material for publication: *SHELXL97*.

## Supplementary Material

Crystal structure: contains datablock(s) global, I. DOI: 10.1107/S1600536812029339/mw2074sup1.cif

Structure factors: contains datablock(s) I. DOI: 10.1107/S1600536812029339/mw2074Isup2.hkl

Supplementary material file. DOI: 10.1107/S1600536812029339/mw2074Isup3.cml

Additional supplementary materials:  crystallographic information; 3D view; checkCIF report

## Figures and Tables

**Table 1:** Hydrogen-bond geometry (Å, °)

*D*—H⋯*A*	*D*—H	H⋯*A*	*D*⋯*A*	*D*—H⋯*A*
N2—H1*C*⋯N1^i^	0.89	1.90	2.776 (2)	169
O1—H1*B*⋯O1^ii^	0.86	1.52	2.378 (2)	176
C1—H1*A*⋯O2^iii^	0.96	2.41	3.278 (2)	150
C8—H8*A*⋯O1^iv^	0.96	2.56	3.190 (2)	124
C9—H9*A*⋯O2^v^	0.96	2.32	3.1903 (19)	150

Symmetry codes: (i) 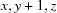; (ii) 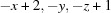; (iii) 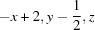; (iv) 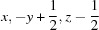; (v) 
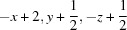
.

**Fig. 1.**
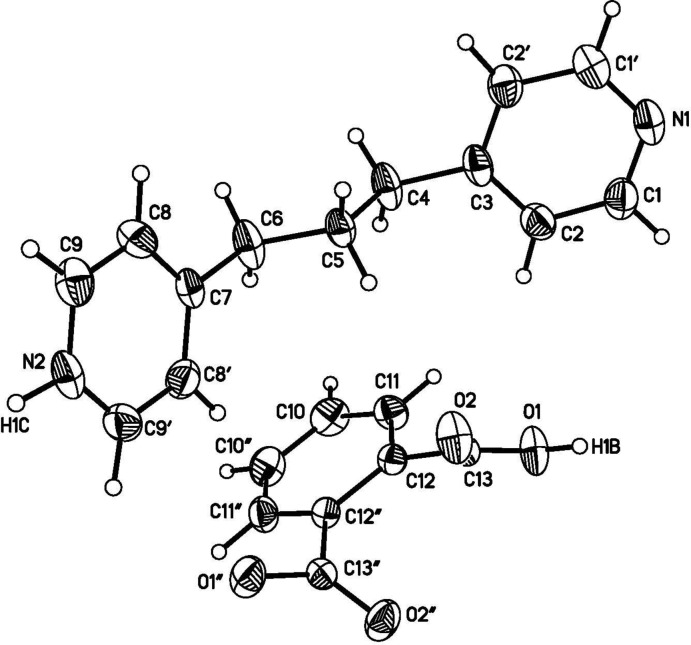
Perspective view of a cation-anion pair in the title compound. Displacement ellipsoids are drawn at 40% probability level. H atoms are presented as a small spheres of arbitrary radius. Only one location is shown for the disordered H1B atom Symmetry code: (') x, y, -z+0.5; (") x, -y+0.5, -z+1.

**Fig. 2.**
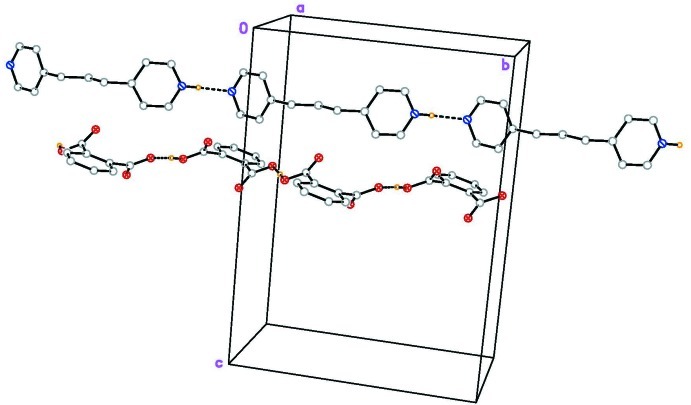
One dimensional chains through O—H···O and N—H···O hydrogen bonds along [0 1 0]. Color key: C = gray, H = orange, N = blue, O = red.

## supplementary crystallographic information

### Comment

Co-crystals have been proven particularly successful as functional materials with applications in pharmaceuticals, molecular electronics, optical applications, and synthetic organic chemistry (Schultheiss & Newman 2009; Sarma *et al.*, 2011). For any given two chemical partners it is always possible to obtain more than one crystalline solid due to the differences in stoichiometries or supramolecular synthons (Callear *et al.*, 2010). The idea of engineering co-crystals serves the purpose of building large solid-state structures without the hassles of covalent synthesis. The synthon that is formed between carboxylic acids and pyridine moieties is one of the most exploited synthon for designing co-crystals (Braga *et al.*, 2011). In this contribution, we present the crystal structure of the phthalic acid and 1,3-bis(4-pyridyl)propane (bpp) co-crystal.

In the title compound the bppH^+^ cation lies on a mirror plane while the 2-carboxybenzoate anion lies on a two-fold axis (Fig. 1). The anions are linked into chains parallel to the [010] direction by strong O—H···O hydrogen bonds with an O···O distance of 2.378 (2) Å and with the H atom disordered about the intervening inversion centre. The bppH^+^ cations engage in N—H···O hydrogen bonds to forms chains extending along the *b* axis (Fig. 2). Weak C—H···O hydrogen bond interactions between the cationic and anionic chains are responsible for the three-dimensional framework assembly (Table 1).

### Experimental

1:1 molar quantities of phthalic acid (0.166 g, 1 mmol) and 1,3-bis(4-pyridyl)propane (0.198 g, 1 mmol) were dissolved in a water-methanol (1:1) mixture (15 mL) and the solution stirred for 10 min. After slow evaporation of the solution for one week at room temperature, colorless block crystals suitable for X-ray diffraction were obtained.

### Refinement

All H atoms were located in a difference map. Those attached to C and N were adjusted to give C—H = 0.97 - 0.98 Å and N—H = 0.89 Å and included as riding contributions with *U*_iso_(H) = 1.2*U*_eq_(C, N). Careful inspection of a difference map in the region between the oxygen atoms of the anions flanking the inversion centre indicated a significant elongation of the density along the line joining the two oxygen atoms suggesting a disorder of this hydrogen about the centre. This atom was placed in the best location indicated by the difference map (O—H = 0.86 Å) and included as a riding contribution with *U*_iso_(H) = 1.2*U*_eq_(O).

### Crystal data

**Table:**

C_13_H_15_N_2_^+^·C_8_H_5_O_4_^−^	*F*(000) = 768
*M**_r_* = 364.39	*D*_x_ = 1.433 Mg m^−^^3^
Orthorhombic, *P**b**c**m*	Mo *K*α radiation, λ = 0.71073 Å
Hall symbol: -P 2c 2b	Cell parameters from 10355 reflections
*a* = 7.5950 (15) Å	θ = 3.0–27.4°
*b* = 12.822 (3) Å	µ = 0.10 mm^−^^1^
*c* = 17.340 (4) Å	*T* = 295 K
*V* = 1688.6 (6) Å^3^	Block, colorless
*Z* = 4	0.35 × 0.24 × 0.21 mm

### Data collection

**Table:**

Rigaku R-AXIS RAPID CCD diffractometer	2097 independent reflections
Radiation source: fine-focus sealed tube	1468 reflections with *I* > 2σ(*I*)
Graphite monochromator	*R*_int_ = 0.039
ω scans	θ_max_ = 28.8°, θ_min_ = 3.1°
Absorption correction: multi-scan (*ABSCOR*; Higashi, 1995)	*h* = −10→10
*T*_min_ = 0.761, *T*_max_ = 0.865	*k* = −16→16
16479 measured reflections	*l* = −22→22

### Refinement

**Table:**

Refinement on *F*^2^	Secondary atom site location: difference Fourier map
Least-squares matrix: full	Hydrogen site location: inferred from neighbouring sites
*R*[*F*^2^ > 2σ(*F*^2^)] = 0.042	H-atom parameters constrained
*wR*(*F*^2^) = 0.118	*w* = 1/[σ^2^(*F*_o_^2^) + (0.0519*P*)^2^ + 0.2824*P*] where *P* = (*F*_o_^2^ + 2*F*_c_^2^)/3
*S* = 1.14	(Δ/σ)_max_ = 0.001
2097 reflections	Δρ_max_ = 0.25 e Å^−^^3^
134 parameters	Δρ_min_ = −0.24 e Å^−^^3^
0 restraints	Extinction correction: *SHELXL97* (Sheldrick, 2008), Fc^*^=kFc[1+0.001xFc^2^λ^3^/sin(2θ)]^-1/4^
Primary atom site location: structure-invariant direct methods	Extinction coefficient: 0.009 (2)

### Special details

**Table:**

Geometry. All esds (except the esd in the dihedral angle between two l.s. planes) are estimated using the full covariance matrix. The cell esds are taken into account individually in the estimation of esds in distances, angles and torsion angles; correlations between esds in cell parameters are only used when they are defined by crystal symmetry. An approximate (isotropic) treatment of cell esds is used for estimating esds involving l.s. planes.
Refinement. Refinement of F^2^ against ALL reflections. The weighted R-factor wR and goodness of fit S are based on F^2^, conventional R-factors R are based on F, with F set to zero for negative F^2^. The threshold expression of F^2^ > 2s˘F^2^) is used only for calculating R-factors(gt) etc. and is not relevant to the choice of reflections for refinement. R-factors based on F^2^ are statistically about twice as large as those based on F, and R- factors based on ALL data will be even larger.

### Fractional atomic coordinates and isotropic or equivalent isotropic displacement parameters (Å^2^)

**Table:**

	*x*	*y*	*z*	*U*_iso_*/*U*_eq_	Occ. (<1)
N1	0.8736 (2)	−0.21026 (13)	0.2500	0.0453 (5)	
N2	0.8483 (2)	0.57374 (13)	0.2500	0.0429 (5)	
H1C	0.8714	0.6418	0.2500	0.051*	
C1	0.8371 (2)	−0.16092 (12)	0.31554 (11)	0.0493 (4)	
H1A	0.8639	−0.1968	0.3627	0.059*	
C2	0.7584 (2)	−0.06488 (12)	0.31769 (10)	0.0467 (4)	
H2A	0.7353	−0.0339	0.3671	0.056*	
C3	0.7146 (2)	−0.01559 (14)	0.2500	0.0384 (5)	
C4	0.6189 (3)	0.08572 (14)	0.2500	0.0479 (6)	
H4A	0.5447	0.0900	0.2954	0.057*	
C5	0.7251 (3)	0.18449 (13)	0.2500	0.0357 (5)	
H5A	0.8000	0.1860	0.2953	0.043*	
C6	0.6092 (3)	0.27893 (15)	0.2500	0.0498 (6)	
H6A	0.5345	0.2759	0.2047	0.060*	
C7	0.6985 (2)	0.38213 (14)	0.2500	0.0341 (5)	
C8	0.7398 (2)	0.43148 (11)	0.18191 (10)	0.0440 (4)	
H8A	0.7151	0.3996	0.1330	0.053*	
C9	0.8148 (2)	0.52775 (12)	0.18304 (10)	0.0473 (4)	
H9A	0.8420	0.5663	0.1371	0.057*	
O1	0.86718 (15)	0.04809 (8)	0.50729 (7)	0.0502 (3)	
H1B	0.9617	0.0127	0.4997	0.060*	0.50
O2	0.97024 (14)	0.17373 (8)	0.43243 (7)	0.0512 (4)	
C10	0.3974 (2)	0.19800 (13)	0.48994 (11)	0.0490 (4)	
H10A	0.2898	0.1610	0.4801	0.059*	
C11	0.54838 (18)	0.14650 (12)	0.48015 (9)	0.0408 (4)	
H11A	0.5480	0.0747	0.4645	0.049*	
C12	0.70198 (17)	0.19725 (10)	0.48994 (8)	0.0302 (3)	
C13	0.86229 (17)	0.13822 (10)	0.47497 (8)	0.0324 (3)	

### Atomic displacement parameters (Å^2^)

**Table:**

	*U*^11^	*U*^22^	*U*^33^	*U*^12^	*U*^13^	*U*^23^
N1	0.0325 (9)	0.0294 (8)	0.0741 (14)	0.0014 (7)	0.000	0.000
N2	0.0304 (9)	0.0241 (8)	0.0741 (14)	−0.0003 (7)	0.000	0.000
C1	0.0470 (9)	0.0382 (8)	0.0628 (11)	0.0019 (7)	−0.0092 (8)	0.0035 (7)
C2	0.0489 (9)	0.0369 (8)	0.0542 (10)	0.0009 (7)	−0.0054 (8)	−0.0073 (7)
C3	0.0283 (9)	0.0228 (9)	0.0640 (14)	−0.0036 (8)	0.000	0.000
C4	0.0332 (11)	0.0247 (10)	0.0857 (18)	−0.0003 (8)	0.000	0.000
C5	0.0301 (9)	0.0243 (9)	0.0526 (13)	−0.0011 (8)	0.000	0.000
C6	0.0323 (10)	0.0260 (10)	0.0910 (19)	0.0005 (8)	0.000	0.000
C7	0.0268 (9)	0.0228 (9)	0.0526 (13)	0.0027 (7)	0.000	0.000
C8	0.0487 (9)	0.0393 (8)	0.0439 (9)	0.0007 (7)	0.0065 (7)	−0.0084 (7)
C9	0.0466 (9)	0.0405 (8)	0.0549 (10)	0.0019 (7)	0.0135 (8)	0.0077 (7)
O1	0.0505 (6)	0.0353 (5)	0.0648 (8)	0.0142 (5)	0.0181 (6)	0.0157 (5)
O2	0.0420 (6)	0.0424 (6)	0.0693 (8)	0.0060 (5)	0.0224 (6)	0.0121 (5)
C10	0.0287 (7)	0.0587 (10)	0.0596 (11)	−0.0085 (6)	−0.0012 (7)	0.0015 (8)
C11	0.0390 (8)	0.0348 (7)	0.0484 (9)	−0.0082 (6)	0.0007 (7)	−0.0002 (7)
C12	0.0297 (7)	0.0289 (7)	0.0321 (7)	−0.0015 (5)	0.0009 (5)	0.0022 (5)
C13	0.0340 (7)	0.0279 (6)	0.0354 (7)	−0.0004 (5)	0.0030 (6)	−0.0020 (6)

### Geometric parameters (Å, º)

**Table:**

N1—C1	1.3299 (19)	C6—H6A	0.9701
N1—C1^i^	1.3299 (19)	C7—C8^i^	1.3758 (19)
N2—C9	1.3269 (19)	C7—C8	1.3758 (19)
N2—C9^i^	1.3270 (19)	C8—C9	1.360 (2)
N2—H1C	0.8900	C8—H8A	0.9600
C1—C2	1.370 (2)	C9—H9A	0.9600
C1—H1A	0.9600	O1—C13	1.2849 (17)
C2—C3	1.3740 (19)	O1—H1B	0.8600
C2—H2A	0.9601	O2—C13	1.1931 (16)
C3—C2^i^	1.3741 (19)	C10—C11	1.334 (2)
C3—C4	1.489 (3)	C10—C10^ii^	1.379 (3)
C4—C5	1.501 (3)	C10—H10A	0.9600
C4—H4A	0.9701	C11—C12	1.3465 (19)
C5—C6	1.497 (3)	C11—H11A	0.9599
C5—H5A	0.9701	C12—C12^ii^	1.397 (3)
C6—C7	1.487 (3)	C12—C13	1.4569 (18)
			
C1—N1—C1^i^	117.43 (19)	C8^i^—C7—C8	118.23 (18)
C9—N2—C9^i^	122.11 (18)	C8^i^—C7—C6	120.88 (9)
C9—N2—H1C	118.3	C8—C7—C6	120.88 (9)
C9^i^—N2—H1C	118.3	C9—C8—C7	120.05 (15)
N1—C1—C2	122.82 (17)	C9—C8—H8A	118.8
N1—C1—H1A	117.2	C7—C8—H8A	121.1
C2—C1—H1A	120.0	N2—C9—C8	119.76 (16)
C1—C2—C3	119.74 (16)	N2—C9—H9A	117.1
C1—C2—H2A	118.4	C8—C9—H9A	123.1
C3—C2—H2A	121.8	C13—O1—H1B	115.6
C2—C3—C2^i^	117.37 (18)	C11—C10—C10^ii^	120.74 (9)
C2—C3—C4	121.30 (9)	C11—C10—H10A	117.7
C2^i^—C3—C4	121.30 (9)	C10^ii^—C10—H10A	121.5
C3—C4—C5	118.30 (17)	C10—C11—C12	119.30 (14)
C3—C4—H4A	109.4	C10—C11—H11A	120.5
C5—C4—H4A	105.3	C12—C11—H11A	120.1
C6—C5—C4	111.53 (16)	C11—C12—C12^ii^	119.96 (9)
C6—C5—H5A	109.2	C11—C12—C13	116.78 (12)
C4—C5—H5A	109.4	C12^ii^—C12—C13	123.20 (7)
C7—C6—C5	116.88 (17)	O2—C13—O1	126.37 (13)
C7—C6—H6A	107.6	O2—C13—C12	119.09 (12)
C5—C6—H6A	108.1	O1—C13—C12	114.43 (12)
			
C1^i^—N1—C1—C2	2.7 (3)	C6—C7—C8—C9	177.27 (16)
N1—C1—C2—C3	−0.3 (3)	C9^i^—N2—C9—C8	1.7 (3)
C1—C2—C3—C2^i^	−2.1 (3)	C7—C8—C9—N2	0.1 (2)
C1—C2—C3—C4	176.15 (16)	C10^ii^—C10—C11—C12	−0.2 (3)
C2—C3—C4—C5	90.90 (17)	C10—C11—C12—C12^ii^	−0.1 (3)
C2^i^—C3—C4—C5	−90.90 (17)	C10—C11—C12—C13	−177.23 (15)
C3—C4—C5—C6	180.0	C11—C12—C13—O2	128.23 (16)
C4—C5—C6—C7	180.0	C12^ii^—C12—C13—O2	−48.8 (2)
C5—C6—C7—C8^i^	−90.46 (16)	C11—C12—C13—O1	−48.27 (18)
C5—C6—C7—C8	90.46 (16)	C12^ii^—C12—C13—O1	134.66 (18)
C8^i^—C7—C8—C9	−1.8 (3)		

Symmetry codes: (i) *x*, *y*, −*z*+1/2; (ii) *x*, −*y*+1/2, −*z*+1.

### Hydrogen-bond geometry (Å, º)

**Table:**

*D*—H···*A*	*D*—H	H···*A*	*D*···*A*	*D*—H···*A*
N2—H1*C*···N1^iii^	0.89	1.90	2.776 (2)	169
O1—H1*B*···O1^iv^	0.86	1.52	2.378 (2)	176
C1—H1*A*···O2^v^	0.96	2.41	3.278 (2)	150
C8—H8*A*···O1^vi^	0.96	2.56	3.190 (2)	124
C9—H9*A*···O2^vii^	0.96	2.32	3.1903 (19)	150

Symmetry codes: (iii) *x*, *y*+1, *z*; (iv) −*x*+2, −*y*, −*z*+1; (v) −*x*+2, *y*−1/2, *z*; (vi) *x*, −*y*+1/2, *z*−1/2; (vii) −*x*+2, *y*+1/2, −*z*+1/2.
